# Testing the Digital Health Literacy Instrument for Adolescents: Cognitive Interviews

**DOI:** 10.2196/17856

**Published:** 2021-03-15

**Authors:** Eunhee Park, Misol Kwon

**Affiliations:** 1 School of Nursing University at Buffalo Buffalo, NY United States

**Keywords:** adolescent, digital health literacy, ehealth literacy, cognitive interview

## Abstract

**Background:**

Despite the increasing number of youth seeking health information on the internet, few studies have been conducted to measure digital health literacy in this population. The digital health literacy instrument (DHLI) is defined as a scale that measures the ability to operate digital devices and read and write in web-based modes, and it assesses seven subconstructs: operational skills, navigation skills, information searching, evaluating reliability, determining relevance, adding self-generated content to a web-based app, and protecting privacy. Currently, there is no validation process of this instrument among adolescents.

**Objective:**

This study aims to explore the usability and content validity of DHLI.

**Methods:**

Upon the approval of institutional review board protocol, cognitive interviews were conducted. A total of 34 adolescents aged 10-18 years (n=17, 50% female) participated in individual cognitive interviews. Two rounds of concurrent cognitive interviews were conducted to assess the content validity of DHLI using the *thinking aloud* method and probing questions.

**Results:**

Clarity related to unclear wording, undefined technical terms, vague terms, and difficult vocabularies was a major issue identified. Problems related to potentially inappropriate assumptions were also identified. In addition, concerns related to recall bias and socially sensitive phenomena were raised. No issues regarding response options or instrument instructions were noted.

**Conclusions:**

The initial round of interviews provided a potential resolution to the problems identified with comprehension and communication, whereas the second round prompted improvement in content validity. Dual rounds of cognitive interviews provided substantial insights into survey interpretation when introduced to US adolescents. This study examined the validity of the DHLI and suggests revision points for assessing adolescent digital health literacy.

## Introduction

### Background

According to the recent report by the International Telecommunications Union, the majority of the world population (93%) resides within reach of mobile broadband (or internet) service [1]. Within this population, it is estimated that 53.6% people–approximately 4.1 billion people—are connected to the internet and have internet access at home [1]. In recent years, digital devices such as mobile phones, tablets, and portable computers have become essential tools in health care. Digital devices have become a medium for improving and facilitating health and well-being, and providing access to health care services for individuals and across populations [2]. The public uses these devices to obtain health-related information on the web and communicate with people using email and social media platforms [3]. In addition, web- and app–based tools aid digital devices in becoming increasingly resourceful in delivering health education and interventions, and empowering users [4]. Given the growth of technology, digital technology-based interventions provide opportunities to improve access to care and create positive health outcomes by enhancing patient engagement and self-management skills using tailored programs and interactive features [5,6].

In particular, youth spend significant time using digital devices such as mobile phones and portable tablets, not only in their daily lives but also for obtaining health-related information and participating in social media for peer support and self-care [3,7,8]. Adolescents in our era are *digital natives* who are comfortable with and attracted to technology and the use of digital devices. It has been reported that teens have a high demand for and openness to the use of digital devices for their health and self-care behavior [8-10]. However, despite general knowledge, they still need to be educated about the specific use of these devices, especially in terms of understanding and deciphering the surplus of content on the web. Thus, it is important to consider digital health literacy and provide proper education and interventions to enhance skills in maneuvering and making decisions based on the information found on the web; this is expected to improve the ability to appropriately use digital devices in the context of health, as they are closely related to mobile health usability and may influence the outcomes of digital-based health care intervention and education. Subsequently, as a high percentage of information is provided on the web for youth—and they are inclined to use digital modes of communication—, such efforts would influence young people’s health behaviors and create positive outcomes [11-15].

To capture how youth interpret digital health information and use multiple digital devices for health, the notion of digital health literacy is most frequently defined as the ability to find and understand health-related information on the web and to write and post interactive features on the internet [16]. Digital health literacy is similar to the concept of *eHealth literacy*, which refers to the ability to read and write in web-based modes requiring multiple components from information, science, health, media, and computer literacy [17-20]. Unlike eHealth literacy, which merely focuses on the ability to read and write information on the web based on *health 1.0 skills*, digital health literacy expands these concepts by including the skills needed to write and post health-related messages on the web based on *health 2.0 skills* [17]. The terms *health 1.0* and *health 2.0* originated from the concept of *web 2.0* in the domain of health, and *health 2.0* indicates advanced technology involving patient empowerment and involvement, sharing information, and social networking [21]. This is important given the development of social media features and web-based discussion threads, which not only require the ability to search for health-related information, understand the information, and apply it appropriately but also to write and post their own information on the web [22].

Given the importance of understanding and applying health information on the web, health researchers have made efforts to develop instruments to assess digital health literacy. However, most of these instruments have only focused on specific aspects of digital health literacy, particularly with regard to health 1.0 skills. For instance, Rapid Estimate of Adolescent Literacy in Medicine-Teen [23] assesses the literacy level to correctly pronounce health-specific terms but does not include an assessment of the understanding of those terms. Newest Vital Sign focuses on understanding numeric information in the context of health [24]. The eHealth Literacy Scale assesses young people’s perceived health literacy regarding finding, assessing, and understanding electronic health information but does not assess the ability to write messages or posts on the web or manage digital devices [17,25]. Digital Health Literacy Assessment Tool assesses adolescents’ ability to find and assess health-related information on the web [26,27].

### Objectives

Considering the limitations of the existing instruments, the digital health literacy instrument (DHLI) [16] assesses an expanded concept of digital health literacy, assessing seven skills: (1) operation skills, (2) navigation skills, (3) information searching, (4) evaluating reliability, (5) determining relevance, (6) adding self-generated content, and (7) protecting privacy, which were identified as necessary skills needed for health-related internet use [16,28]. This instrument, designed for adult users, offers many components relevant to the assessment of digital health literacy based on health 2.0 skills. We will use a DHLI, which is an instrument developed to assess digital health literacy, as a model for our research focused on the adolescent population. The scale comprises 21 self-reported items for adults. It was originally developed in Dutch and translated into English, and the developers conducted cognitive interviews to ensure its validity in adults [29,30]. Thus, its reliability and validity are supported for the Dutch adult population [16]. The internal consistency of the total scale (Cronbach α=.87) and the subscales (Cronbach α=.70-.89) was supported, but the value for the privacy protection subscale (Cronbach α=.57) was low. A test-retest analysis was conducted, and intraclass correlation coefficients for the total scale and subscales ranged from .49 to .81 [16]. Confirmatory factor analysis supported the 7 constructs. Correlations with significant associations were reported for age, education, internet use, health-related internet use, self-reported health status, health literacy, and eHealth to support validity of the instrument [16].

Although digital health literacy is an important concept, there are limited instruments available for adolescents. The lack of existing instruments that effectively assess adolescents’ digital health literacy has led to a limited understanding of young people’s comprehension skills and the ability to use the internet and digital devices for health-related purposes [27]. Unfortunately, this reduces the chances of successful results of prevalent adolescent health education and health promotion programs with digital devices. On the basis of these concerns, we aim to explore usability and assess the validity of DHLI using cognitive interviews among US adolescents (aged 10-18 years). Cognitive interviews are an essential step in ensuring the validity of the questionnaire development process. Cognitive interviews provide a chance to find sources of errors in survey research and allow for exploration of the understanding of the questionnaire among the target population [28]. Thus, the findings of this study will contribute to modifying DHLI and tailoring the instruments targeting adolescents in the United States.

## Methods

### Research Design

After approval from the affiliated university, cognitive interviews were conducted. Parent consent forms and minor assent forms were obtained. Concurrent cognitive techniques have been used [31]. Two interviewers were trained for the cognitive interviews with adolescents. The first author, who had experience conducting cognitive interviews related to previous works, provided the essential literature on cognitive interviews, including books, example articles, and an interview guide, to the other research team members (2 interviewers). The research team members read the selected books and articles, after which the first author guided the interview process step by step. We used the training procedure suggested by Willis [32]. The research team members met regularly (>3 times) during the 2 weeks of training sessions until the interviewers felt comfortable with and confident about the procedures and were able to conduct the interview on their own. The teach-back method, in which a trained interviewer teaches and prompts the training interviewer as guided, was used, allowing for a standardized process. After each interview, we listened to each other’s interview audio recordings and provided feedback and suggestions for improvement when necessary. In addition, we met on a weekly basis and discussed elements of the interview process, such as clarifying procedures, resolving any obstacles, and discussing probing questions. The interview process consisted of one-on-one individual interviews, and the audio was recorded.

### Participants

A total of 34 adolescents completed the interviews. The sample was composed of 50% (n=17) females and 50% (n=17) males aged between 10 and 18 years. The ethnic background included White Americans (n=15, 44%), African Americans (n=11, 32%), Latino (n=3, 9%), and Asian Americans (n=5, 15%). The mean age of the participants was 13.47 (SD 2.39) years. Table 1 provides detailed demographic information of the respondents. Participants were recruited via flyers posted during summer at the youth community centers and universities where adolescents often visit for summer camp programs.

**Table 1:** Demographics of the recruited adolescents (N=34).

Variables		Participants, n (%)
**Gender, n (%)**		
	Female	17 (50)
	Male	17 (50)
**Race, n (%)**		
	White American	15 (44)
	Black or African American	11 (32)
	Latino or Hispanic	3 (9)
	Asian American	5 (15)
Age (years), mean (SD)		13.47 (2.39)
**School achievement, n (%)**		
	Much better than average	3 (9)
	Better than average	16 (47)
	Average	14 (41)
	Much worse than average	1 (3)
**Literacy level, n (%)**		
	Much better than average	8 (24)
	Better than average	13 (38)
	Average	13 (38)
**Health insurance type, n (%)**		
	Private	4 (12)
	Government-sponsored	7 (21)
	Does not know	23 (68)
**Free lunch at school, n (%)**		
	Yes	21 (62)
	No	13 (38)

We used a quota sampling approach for sample selection [32,33]. We aimed to recruit a similar proportion of male and female participants (50% each). Recruitment also accounted for the racial and ethnic composition of the US population. For example, according to the census data from 2018, the US population was 61% White, 13% Black or African American, 17% Latino or Hispanic (17%), 5% Asian, 1% American Indian or Alaska Native, and 1% Native Hawaiian or other Pacific Islander. We attempted to recruit participants corresponding to the number of participants for each racial and ethnic group. Among our participants, there was a slightly higher percentage of Black or African Americans and Asian Americans and a lower percentage of White Americans and Latino or Hispanics than we had intended. We could not recruit participants from American Indian, Alaska Native, Native Hawaiian, or other Pacific Islander groups. In addition, we recruited from all age groups, resulting in at least 3% of all participants in each age group from 10 to 18 years.

### Data Collection

Each face-to-face interview was conducted in English and lasted for 30 minutes to 1 hour. Before each interview, the researcher explained the purpose and process of the interview to the participants. In the first round, 22 interviews were conducted. On the basis of the findings from the first round of interviews, the research team analyzed the data and made a suggested revision to the instruments. In the second round, we conducted 12 interviews with the original and revised items. According to Willis [33], 12-15 is a good target number of interviews for each round. However, we felt the need to include more than 15 interviews to ensure a diverse population of adolescents, especially those of various ages [33]. Therefore, we had 22 participants. We stopped recruiting participants when saturated themes emerged, which indicated that no more new issues or themes arose during the interviews.

The participants read each item and were encouraged to think aloud with regard to each item. The researcher then asked probing questions based on the previous literature [32,34]. All interview files were transcribed verbatim.

### Data Analysis

The researchers listened to the audio recordings, and the transcribed data were coded based on the Miles and Huberman approach [35]. Using the Miles and Huberman approach, we followed a deductive approach to analyze the interview data. We formulated a clear research question and used the theoretical framework to guide the study. This approach was chosen because of the reasoning that the nature of our study would benefit from this analytic approach with the existing framework and facilitate the assessment of the instrument’s content validity in a more structured way. We initially conducted descriptive coding by labeling each unit of meaning related to specific issues raised for each questionnaire item. Guided by the Willis [32] framework, we established the following main categories: reading, instruction, clarity, assumptions, knowledge or memory, sensitivity or bias, response categories, and other problems [32-34]. The following categories and subdomains of the Willis [32] framework (Table 2) were used to ensure the instrument’s content validity when organizing the codes. In addition, the framework provides clear categories with subdomains, which are important for ensuring content validity in a survey development process based on user feedback from existing questionnaires. The authors checked for any emergent codes in the data, but none were found.

**Table 2:** Results of cognitive interviews.

Category and subdomains		Original item	Revised items
**Clarity**			
	**Wording**		
		5a. Do you find it difficult to judge who can read along?	Do you find it difficult to *know who will read the message*?^a^
	**Technical terms**		
		1c. Do you use the buttons or links and hyperlinks on websites?	Do you use the buttons or links on websites?
		4. When typing a message (eg, to your doctor, on a forum or on social media such as Facebook or Twitter) how easy or difficult is it for you to...	When typing a message *online* (eg, to your doctor; on a *website*; *blog*; or on social media such as Facebook, Twitter, *Snapchat*, or *Instagram*) how easy or difficult is it for you to...
		5. When you post a message on a public forum or social media, how often...	When you *write* a message on a *website*, *blog*, or social media, how often...
	**Vague**		
		2e. Decide whether the information is written with commercial interests (eg, by people trying to sell a product).	Decide whether the information is written *for advertisement* (eg, by people trying to sell a product).
		2g. Decide if the information you found is applicable to you.	Decide if the information you *find relates* to you (eg, *school homework*, *exercise*, and *eating habits*).
		2h. Apply the information you found in your daily life.	Apply the information you *find* in your daily life (eg, *school homework*, *exercise*, and *eating habits*).
		5b. Do you (intentionally or unintentionally) share your own private information (eg, name or address)?	Do you share your private information (eg, name or address, *location*, and *school information*)?
		5c. Do you (intentionally or unintentionally) share some else’s private information?	Do you share *someone* else’s private information (eg, name or address, *location*, and *school information*)?
	**Difficult vocabulary**		
		2b. Use the proper words or search query to find the information you are looking for.	Use the *keywords* or search *term* to find the information you are looking for.
		2d. Decide whether the information is reliable or not.	Decide whether the information is *trustworthy* or not.
		2g. Decide if the information you found is applicable to you.	Decide if the information you found *relates* to you (eg, *school homework*, *exercise*, and *eating habits*).
		4a. Clearly formulate your question or health-related worry.	Clearly *write* your question or health-related worry.
**Assumption**			
	**Inappropriate assumptions**		
		1a. Use the keyboard of a computer (eg, to type words).	Use the keyboard of a computer, or a *tablet*, or a *phone* (eg, to type words).
		1b. Use the mouse (eg, to put the cursor in the right field or to click).	Use the mouse or a *touchpad* (eg, to put the cursor in the right field or to click).
		4. When typing a message (eg, to you doctor, on a forum or on social media such as Facebook or Twitter) how easy or difficult is it for you to...	When typing a message *online* (eg, to your doctor; on a *website*; *blog*; or on social media such as Facebook, Twitter, *Snapchat*, or *Instagram*) how easy or difficult is it for you to...
		5. When you post a message on a public forum or social media, how often...	When you *write* a message on a *website*, *blog*, or social media, how often...
**Knowledge**			
	Recall	2h. Apply the information you found in your daily life.	Apply the information you *find* in your daily life (eg, *school homework*, *exercise*, and *eating habits*).
**Sensitivity/bias**			
	**Socially acceptable**		
		5b. Do you (intentionally or unintentionally) share your own private information (eg, name or address)?	Do you share your private information (eg, name or address, *location*, and *school information*)?
		5c. Do you (intentionally or unintentionally) share some else’s private information?	Do you share *someone* else’s private information (eg, name or address, *location*, and *school information*)?

^a^Please see the text in italics for the revised phrases.

## Results

### Overview of the Results

We conducted 34 cognitive interviews across 2 rounds to evaluate clarity, assumptions, knowledge, and sensitivity or bias in the items. Subthemes that emerged from the 4 categories included wording, technical terms, vagueness, difficult vocabulary, inappropriate assumptions, recall, and being socially acceptable. The initial round of interviews provided a potential resolution to comprehension and communication problems. We determined the need to propose a modification when participants repeatedly brought up similar issues during the initial round. Subsequently, the research team modified the items using collaborative clinical judgment. We had no specific quantitative cut-off points (eg, the minimum percentage of participants who raised each issue) because no such numbers are suggested in the literature, given that most cognitive interviews involve a small number of participants. When the minimum percentage of the participants who raised the same issue during the initial round (n=22) for the modified items was calculated, it was 18% (n≥4). During the modification phase, the researchers reflected on the participants’ responses and suggestions. For instance, modifications were made by adding examples that were reflective and inclusive of current technological devices (eg, mouse vs mouse and touchpad). Most issues that indicated the need for improvement in the areas of clarity, assumptions, and knowledge emerged during the first round, whereas the second round provided a more confirmatory process, prompting improvement in content validity. Examples of changes for each category are presented in Table 2.

### Clarity

#### Wording

Clarity of the items requires wording that is most appropriate to best explain a question being asked. We defined problematic wording as that which is seemingly lengthy, awkward, ungrammatical, or contains complicated syntax for the respondents. For instance, half of the respondents in round 1 (11/22, 50%) expressed awkwardness in the word *to judge* in the item 5a: “When you post a message on a public forum or social media, how often do you find it difficult to judge who can read along?”

When asked to interpret in their own words, many found the item difficult to comprehend and stated that it did not make sense even after reading it several times. A respondent stated:

I think I'm just getting confused at do you find it difficult to judge the community along – who's your – what is it asking?

In addition, participants expressed confusion by the phrase *who can read along*. In round 2, we provided 3 options: wordings in the item that remained the same and later ones that replaced *to judge* with *to know* and *to decide*. *To know* was better received by the majority (over 8/12, 65%) of the respondents.

#### Technical Terms

The original items included undefined, unclear, or complex terms that were either redundant or needed more examples that best catered to the adolescent population. Over half of the respondents (13/22, 59%) struggled to define the term *hyperlinks* (item 1c); hence, it was removed. Likewise, several participants (7/22, 32%) had varied definitions of *forum* (item 4), ranging from a *form/document* to a *place where professionals gather*. Hence, the word *forum* was replaced with *website* *or* *blog*. Moreover, the participants (7/22, 32%) found Facebook and Twitter as examples of social media networking websites in the original survey (item 4) to lack relatability and suggested the inclusion of Snapchat and Instagram.

#### Vague

Vagueness can be a linguistic and nonlinguistic mechanism that enables readers to interpret questions based on their own understanding. This leads to interpreting the question in multiple ways, deciding what is to be included or excluded, or being selective based on their own definition, all of which prohibits a clear understanding. In round 1, many respondents (14/22, 64%) verbalized that *commercial interests* would be hard to pinpoint, especially if no examples were to be provided (item [2e]):

When you search the internet for information on health, how easy or difficult is it for you to decide whether the information is written with commercial interests?

eg, by people trying to sell a product

Respondents communicated that either replacing *commercial interest* with *advertisement* or shortening the question to “...do the commercials influence you during web-surfing” would yield more accurate responses.

Similarly, respondents (4/22, 18%) suggested the inclusion of examples that were more specific to children and adolescents to yield better responses. Hence, examples such as *school homework, exercise, eating habits* was added to item 2g and 2h, and *location, school information* were added to item 5b, and 5c, respectively. In round 2, respondents were probed on their confidence in providing an accurate understanding of these items, and they showed a better understanding with these examples. A respondent provided feedback by stating:

I like how you put the examples. I think that really helps. Because someone else can completely understand what that means. I think you just feel like ah like this is all about my homework. All this helped me to choose what food to eat. So I think it was really important for the examples. That really helps.

#### Difficult Vocabulary

Text comprehension can be influenced by the quantity and location of difficult vocabulary in a given statement. Respondents (10/22, 45%) felt that words such as *applicable* (item 2g) and *formulate* (item 4a) could be replaced with easier words. This was especially evident with younger respondents (aged 10-12 years) who did not understand what the words meant but vaguely tried to guess in their own ways. Suggestions were taken into consideration for round 2. With changes in round 2, all participants (n=12) expressed no difficulty in understanding. In addition, the *proper words* described in item 2b were confusing to the respondents (4/22, 18%). This was taken into consideration in round 2, and options were provided that replaced *proper words* with *key words*, *appropriate (key) words*, or *correct (key) words*. All options were provided, and most of the respondents (9/12, 75%) found *key words* to be an easier option for conveying the meaning embedded in the item.

In round 1 of cognitive interviews, several participants (11/22, 50%) indicated that the word *reliable* is understandable, but replacing it with *trustworthy* would be easier to understand, as it is more commonly used by their age group. In addition, more than half of the respondents (12/22, 55%) reported difficulty with the vocabulary—the *query*. Many had difficulty rephrasing the word or the item, as also described by one of the older respondents:

I understand and I know what a search query is. It just throws me off a little bit because it's not a very... it's not commonly used jargon for most people that I interact with. So that threw me off a little bit when you first read it to me.

Aged 18 years, female

The *search query* was then replaced with *term* for round 2, and all respondents (n=12) were able to comprehend the item with ease.

### Assumptions: Inappropriate Assumptions

Survey questions require wording or phrases that do not make assumptions or draw conclusions about the respondent or his or her circumstances. For instance, the original survey of item 1a asks, “How easy or difficult is it for you to use the keyboard of a computer (eg, to type words)*?*” Early in round 1 of cognitive interviews, 18% (4/22) of the respondents suggested adding a computer, tablet, or phone, as teens nowadays increasingly use a phone or tablet more than a computer. When asked to apply the question to the individual, another respondent replied:

I use my fingers to type on the, on my phone, and my tablet, and stuff.

Hence, *touch pad* was added to the original item 1b, “How easy or difficult is it for you to use the mouse (eg, to put the cursor in the right field or to click)?”, and was rephrased to *mouse or touch pad* in round 2. In addition, contrary to the general belief, many younger respondents in the study stated that although they possess a social media account, they do not use them regularly.

### Knowledge: Recall

The ability to answer survey questions appropriately and precisely requires respondents to be able to recall instances in their lives to answer the question. For instance, 18% (4/22) of the respondents found the original survey item 2h, “When you search the internet for information on health, how easy or difficult is it for you to apply the information you found in your daily life?” to be difficult to answer, as a respondent quotes:

I feel like it's asking a lot because we might not encounter information one day but if you do it a week from now, you still would remember it so you could use it later, so it's not really, you can't really answer the question because you don't know until you run across the information, like that's really a good fact. You remember it and you use it later on.

This item was revised to read “apply the information you find in your daily life” by changing *found* to *find*. This emphasized perceived real-life applicability, as opposed to the actual application, to reduce recall bias and enhance the item’s validity. However, we were unable to gather feedback on this change during the second round because we came up with this idea after all interviews.

In addition to having difficulty defining what *daily* entails, respondents felt that adding examples that pertain to the question could help them recall better and more quickly. In round 2, we added “(eg, school homework, exercise, eating habits)” at the end of item 2h.

### Sensitivity or Bias: Socially Acceptable

We probed the respondents in an effort to understand whether the survey questions were sensitive in nature or wordings implicated bias. In answering the question about the frequency of sharing private information (eg, name or address) unintentionally and intentionally, most of the respondents confidently responded with *never*. However, when probed regarding the information and posts by their friends or themselves in their social media networking websites or apps, several respondents (4/22, 18%) recanted their response from *never* to *sometimes*, which may indicate potential bias and sensitivity in this question. A respondent stated:

You don’t know everybody on social media but majority of people you do know. So like if you say going to the mall then maybe your friend might comment, “I want to come along.” But you don’t know who is seeing it. So it is kind of private but we don’t think of it like that. But like if you really sit down and think about it then you can understand. But if we are typing, we are just like putting it up for our friends to see and not for everybody - but it is social media so everybody is going to see it. Like their name, their age, like what school they go to. But I’ve been seeing a lot of like – because I only know the 8th grades and 9ths so everybody go to different high schools and stuff but so they’ll be like oh such and such guys have been to this school or this school so yeah.

The privacy issue is pertinent when discussing digital health literacy, as youth often forget how much private information about themselves or others they expose or disclose when posting a public message. We suggested deleting *intentionally or unintentionally* from the original item to reduce potential sensitivity or bias related to differences between intentional and unintentional incidences of privacy breeches and clarify the item.

## Discussion

### Principal Findings

This study conducted cognitive interviews on the DHLI originally developed for adults in Dutch to tailor it to adolescents in the United States. Cognitive interviews were conducted to assess content validity and identified issues based on four categories: clarity, assumptions, knowledge or memory, and sensitivity or bias. On the basis of the following issues, we made suggested revisions to the original items and received feedback in the second round of interviews.

Given that this instrument was originally developed for adults in Dutch and translated into English, clarity was the main issue found. We identified difficult vocabularies, technical terms, and wordings and vague expressions, which prevented a clearer understanding of each item. This finding suggests that back-translation may be necessary to ensure cultural validity in translating instruments.

Given that the digital world is rapidly evolving, some terms are likely to be outdated and may not reflect the current trends. For example, the original items include *mouse* and *computer* and did not include *touch pad* or *tablet*, which suggests that this type of instrument needs to reflect the rapidly changing trend of technology development. This has emerged concerning social-media-related questions. Adolescents did not use Facebook as much as other social media networking websites, such as Snapchat or Instagram. As trending websites and apps tend to evolve rapidly, instruments such as DHLI need to be able to reflect fast-changing trends in a timely manner.

Moreover, social media use or web searches may not be considered as frequent activities among some adolescents, particularly the younger population, which could lead to a potential threat to validity. Thus, we may not assume that all adolescents use social media or the internet for health-related purposes. It is also important to provide specific examples that reflect the context or activities familiar to adolescents when developing items, which will make the items more relevant to adolescents. For example, homework, school information, and eating habits would be examples that could be relevant to adolescents and could be added to the items.

### Limitation and Suggestions for Future Research

This study has some limitations. Although we included 34 participants and aimed at diversity (age, sex, and race or ethnicity) in recruitment, the study included a slightly higher percentage of minority groups (African American, Hispanic, and Asian Americans). In addition, all participants were recruited from the same region (an eastern state in the United States), which may have decreased the generalizability of the findings. Future studies may also need to include participants who may not go to school or for whom English may not be their first language.

In addition, we admit that the developmental span varies even among adolescents across age. Thus, further differentiation may need to be considered—altering the questions to younger and older adolescents to consider their developmental stage and contextual factors and to develop more valid instruments.

Although the DHLI comprehensively assesses the skills necessary for digital health literacy, we only used the part of the scale that is based on the self-report. We were not able to tailor and test the 7 performance-based items in this cognitive interview because those items were provided by Dutch websites. This part of the scale is important to provide insightful understanding of digital health literacy by allowing the assessment of participants’ performance level. It will be difficult to use these items as they are, but it will be important to develop this type of performance-based test for adolescents in the United States.

Another limitation is that this study focused on exploring the instrument’s content validity based on cognitive interviews with adolescent users. Thus, based on the feedback we obtained from adolescents, providing quantitative data on the revised instrument’s reliability and construct validity for adolescents is necessary for future studies. In addition, the suggested revisions are based only on adolescents’ feedback on the translated scale. We made considerable efforts to preserve the original items as much as possible because of the validity and reliability established in previous studies. Nonetheless, further modifications may be necessary to enhance the scale’s reliability and validity. For example, the original version of question 5 does not specifically contain the word *health*. Although the item presumes that the respondents know the questionnaire’s purpose and that the item asks about health-related contexts, such an assumption may threaten the scale’s validity. Further improvements might increase the scale’s validity, as might exploring the revised instrument with experts.

### Implications for Practice

In practice, health care professionals who develop interventions using the internet or digital devices may be able to assess digital health literacy levels among adolescents. Thus, the initial level of digital health literacy can be considered in the delivery of such interventions or education, and the program can be tailored based on the level of literacy [36]. This is a necessary step considering that digital device-based interventions have great advantages and have been beneficial for improving health in various contexts and have been particularly helpful given the nature of attractiveness to adolescents.

### Conclusions

This study fills the important gap in research by exploring the validation of a DHLI for adolescents. Digital health literacy is an important skill that needs to be assessed, enhanced, and considered in the digital era. On the basis of cognitive interviews, the validity of the instrument assessing comprehensive digital health literacy skills was tested, and the items for adolescents with suggested revisions were provided (Multimedia Appendix 1). This will allow for the provision of tailored health education and promotion programs based on individual digital health literacy levels, and personalized effort will increase the chances for better health outcomes for this population.

## Appendix

Multimedia Appendix 1 Revised items.

## Abbreviations

DHLI: digital health literacy instrument
