# Supplementary material for: Testing the Digital Health Literacy Instrument for Adolescents: Cognitive Interviews
Source: J Med Internet Res. 2021 Mar 15;23(3):e17856. doi: 10.2196/17856 (PMC8074835; doi:10.2196/17856)
Supplement: Multimedia Appendix 1 [file jmir_v23i3e17856_app1.docx]

| **Original Items** | | **Revised Items** |
| --- | --- | --- |
| **1. How easy or difficult is it for you to…** | | -* |
| a. | use the keyboard of a computer (e.g. to type words)? | use the keyboard of a computer**, or a tablet, or a phone** (e.g. to type words)? |
| b. | use the mouse (e.g. to put the cursor in the right field or to click)? | use the mouse **or a touchpad** (e.g. to put the cursor in the right field or to click)? |
| c. | use the buttons or links and hyperlinks on websites? | use the buttons or links **~~and hyperlinks~~** on websites? |
| **2. When you search the internet for information on health, how easy or difficult is it for you to…** | | - |
| a. | make a choice from all the information you find? | - |
| b. | use the proper words or search query to find the information you are looking for? | use the **key** words or search **term** to find the information you are looking for? |
| c. | find the exact information you are looking for? | do you share some**one** else’s private information (e.g. name or address, **location, school information**)? |
| d. | decide whether the information is reliable or not? | decide whether the information is **trustworthy** or not? |
| e. | decide whether the information is written with commercial interests? (e.g. by people trying to sell a product?) | decide whether the information is written **for advertisement**? (e.g. by people trying to sell a product?) |
| f. | check different websites to see whether they provide the same information? | - |
| g. | decide if the information you found is applicable to you? | decide if the information you ~~found~~ **find relates** to you **(e.g. school homework, exercise, eating habits)**? |
| h. | apply the information you found in your daily life? | apply the information you ~~found~~ **find** in your daily life **(e.g. school homework, exercise, eating habits)**? |
| i. | use the information you found to make decisions about your health (e.g. on nutrition, medication or to decide whether to ask a doctor’s opinion)? | Use the information you ~~found~~ **find** to make decisions about your health (e.g. on nutrition, medication or to decide to whether to ask a doctor’s opinion)? |

| **3. When you search the internet for health information, how often does it happen that…** | | - |
| --- | --- | --- |
| a. | you lose track of where you are on a website or the internet? | - |
| b. | you do not know how to return to a previous page? | - |
| c. | you click on something and get to see something  different than you expected? | - |
| **4. When typing a message (e.g. to you doctor, on a forum or on social media such as Facebook or Twitter) how easy or difficult is it for you to…** | | When typing a message **online** (e.g. to you**r** doctor, on a **website, blog,** or on social media such as Facebook , Twitter, **Snapchat, or Instagram**) how easy or difficult is it for you to… |
| a. | clearly formulate your question or health-related worry? | clearly **write** your question or health-related worry? |
| b. | express your opinion, thoughts, or feelings in writing? | - |
| c. | write your message as such, for people to understand exactly what you mean? | - |
| **You only have to answer the questions below (5 a - c) if you have ever posted a message on social media, such as Facebook or Twitter, a forum, or a (health care) rating site.** | | |
| **5. When you post a message on a public forum or social media, how often…** | | When you **write** a message on a **website, blog**, or social media, how often… |
| a. | do you find it difficult to judge who can read along? | do you find it difficult to **know who will read the message**? |
| b. | do you (intentionally or unintentionally) share your own private information (e.g. name or address)? | do you share your private information (e.g. name or address, **location, school information**)? |
| c. | do you (intentionally or unintentionally) share some else’s private information? | do you share some**one** else’s private information (e.g. name or address, **location, school information**)? |
| Note. -* no change was made | | |
